# Graphene oxide and bacteria interactions: what is known and what should we expect?

**DOI:** 10.1128/msphere.00715-23

**Published:** 2024-01-10

**Authors:** Diliana D. Simeonova, Katrin Pollmann, Alberto Bianco, Didier Lièvremont

**Affiliations:** 1 The Stephan Angeloff Institute of Microbiology, BAS, Atelier Pasteur, Sofia, Bulgaria; 2 Helmholtz-Zentrum Dresden-Rossendorf, Helmholtz Institute Freiberg for Resource Technology, Dresden, Germany; 3 CNRS, Immunology, Immunopathology and Therapeutic Chemistry, UPR 3572, ISIS, University of Strasbourg, Strasbourg, France; 4 Chemistry and Biochemistry of Bioactive Molecules, University of Strasbourg/CNRS, UMR 7177, Strasbourg Institute of Chemistry, Strasbourg, France; University of Michigan, Ann Arbor, Michigan, USA

**Keywords:** graphene oxide, bacteria, biodegradation, PAH degradation, carbon metabolism

## Abstract

Graphene oxide (GO) and graphene-based materials (GBMs) have gained over the last two decades considerable attention due to their intrinsic physicochemical properties and their applications. Besides, a lot of concern regarding the potential toxicity of GBMs has emerged. One of the aspects of concern is the interactions between GBMs and different environmental compartments, especially indigenous microbial and, in particular, bacterial communities. Recent research showed that GO and GBMs impacted bacterial pure culture or bacterial communities; therefore, these interactions have to be further studied to better understand and assess the fate of these materials in the environment. Here, we present our opinion and hypotheses related to possible degradation mechanisms of GO that can be used by environmental bacteria. This work is the first attempt to deduce and summarize plausible degradation pathways of GO, from structurally similar recalcitrant and toxic compounds, such as polyaromatic hydrocarbons.

## OPINION/HYPOTHESIS

### General properties of GBMs

Carbon-based nanomaterials possess exceptional specific mechanical properties such as high tensile strength and elasticity as well as electrical and thermal conductivity, which predetermine their broad use as additives for polymers and coatings in transparent conductive layers or as photovoltaic back-contact material, in nanomedicine as drug delivery systems or even in wastewater treatment for absorption of pharmaceuticals and other pollutants (1, 2). Graphene is a two-dimensional (2D) material made up of a single layer of carbon atoms arranged in a hexagonal lattice. The more diverse graphene-based materials (GBMs) are groups of 2D carbon nanomaterials with up to 10 layers of graphene, which can contain other chemical elements. Within this family, graphene oxide (GO), which bears hydrophilic functional groups, that is, hydroxyls, epoxides on their basal planes and carbonyls, and carboxyls at the carbon sheet edges, is one of the most important graphene derivatives and a modified form of the naturally occurring mineral graphite. Furthermore, GO is the key intermediate for the production of various chemically modified and functionalized GBMs (1, 3).

Unfortunately, the advantages of these materials are also their major deficiency: GO and GBMs are potentially persistent in the environment, and as they are now being produced and used at an increasing rate, their potential biological transformations and degradation by microorganisms are questioned.

### Health and environmental concerns of GO and GBMs

The physicochemical characteristics of these nanomaterials have been demonstrated to have a significant impact on their toxicity, that is, the lateral size and sharpness of the edges, the oxidation state of the material, and functionalization (4). In a recent review (5), extensive toxicological studies performed with GO and some GBMs on diverse organisms, for example, bacteria, planktonic crustaceans, terrestrial invertebrates, water fleas, brine shrimps, fishes, plants, and algae, were examined relative to endpoints such as EC_50_ and LC_50_.

Research on the environmental release of graphene materials and how these materials might enter ecosystems is needed and is ongoing. The quantities released, primarily through industrial processes and product use, will depend on the production and use of GBMs, as well as on the waste disposal practices, and finally on the ability to control and contain these materials during production and application. It is estimated that today’s global demand for graphene alone reaches the kt year^−1^ mark (6). Nevertheless, to date, no study reports the detection of these materials in environmental compartments or focuses on their persistence.

### Bacterial communities and interface with GBMs

Because of their chemical structures, the transformation and degradation of GO and GBMs by microorganisms represent a real challenge.

**Fig 1:**
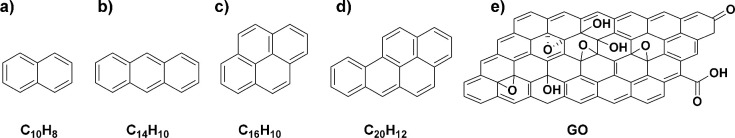
Structural formula of (a) naphthalene, (b) anthracene, (c) pyrene, (d) benzo[a]pyrene, and (e) GO.

Environmental bacteria have not yet been explored in depth for their capacity to interact with these emerging nanomaterials. In a similar way to the polyaromatic hydrocarbons (PAHs), GBMs could constitute a carbon and energy source extremely difficult to assimilate for bacteria. Nonetheless, based on decades of successful studies on PAH bacterial degradation, such as naphthalene, anthracene, pyrene, benzo[a]pyrene (Fig. 1), and many other compounds (7), by bacterial consortia and pure cultures of aerobes or anaerobes, we could reasonably consider that environmental bacteria should be able to interact, transform, and even degrade GO or GBMs.

In 2015, the first hints about the influence of different GBMs and GO on natural environmental bacterial communities were published (8, 9). The use of different environmentally relevant concentrations of GBMs (from 1 ng to 1 mg·kg^−1^ dry soil) induced significant changes in bacterial soil community structures but did not influence the α-diversity of metagenomic libraries (10). This is a confirmation that the enrichment of specific bacterial soil consortia is induced by the addition of carbon-based 2D materials according to the type of graphene used. In addition, a partial convergence of metagenomic communities was observed in the presence of graphite and GO or graphite and other GBMs, which indicates that bacteria have diverse options to cope or interact with different GBMs (10). A recent review focused on the interactions between GBM and microbial communities originating from different environmental compartments, including soil (e.g., paddy, urban, forest, mountain, and grassland), surface waters (e.g., river, lake, and estuary), and sediments, which demonstrates increased interests in these research topics (2). These works provide a good outlook for further studies related to environmental bacteria from different compartments, including those from graphite mines, and their ability to interact or degrade GO and other GBMs, thus opening new frontiers in microbiology and white biotechnology.

### Impact on aerobic bacteria

The predominant part of scientific literature related to interactions between GO and bacteria is based on toxicity studies of graphene, GO, or other GBMs toward a small number of human opportunistic bacterial pathogens, used in medical microbiology (9). Such test strains are *Escherichia coli*, *Pseudomonas aeruginosa*, *Streptococcus aureus*, *Bacillus subtilis*, and a few other well-known Gram-positive and Gram-negative bacterial model strains. However, as human opportunistic pathogens, these bacteria require a relatively narrow range of specific growth conditions. They are growing on rich and complex nutrient media, at an optimal temperature of 37°C and a pH between 6.8 and 7.5. Although useful for medical microbiology, these bacteria and other opportunistic or strictly pathogenic bacteria are not representative of the extensive natural diversity of bacterial life modes on Earth. Thus, for studies on bacterial-GO interactions and bacterial abilities to transform or degrade GBMs, bacteria from different environmental compartments are foremost appropriate. Meanwhile, several bacteria from different environments have been described to interact with GO or graphene materials. In 2018 Qu et al. isolated and identified from soil a novel strain of the bacterial genus *Labrys* sp. WJW that used GO for growth (11). Degradation of the materials was confirmed by atomic force microscopy (AFM) and transmission electron microscopy (TEM) methods with aromatic intermediates identified by gas chromatography-mass spectrometry (GC-MS) analysis. From genomic and proteomic analyses, it was assumed that several peroxidases, oxidoreductases, lyases, and hydrolases were involved in biodegradation. Besides, a new bacterial strain able to grow with naphthalene as the sole carbon source was isolated from the contaminated soil of a graphite mine (12). Further studies proved that it was able to degrade graphite, GO, and reduced GO. Interestingly, the bacteria had different effects on these materials with more defects in reduced GO, indicating that this material was more highly oxidized than graphite. However, bacteria were not further described, and metabolites and metabolism were not studied. An earlier study investigated the degradation of ^14^C-labeled multiwall carbon nanotubes (MWCNTs)—another complex carbon nanomaterial (13). The authors identified a consortium of *Burkholderia kururiensis*, *Delftia acidovorans*, and *Stenotrophomonas* that degraded acid-treated MWCNTs in the presence of an external carbon source, under environmentally relevant conditions. Several polyaromatic compounds were identified as intermediate metabolites.

The chemolithoautotrophic microorganism *Acidithiobacillus ferrooxidans* CFMI-1 was used for the oxidation of graphite to produce GO (14). This strain can mildly oxidize the natural pure graphite and also can bioerode the graphite and produce many few-layer nanosheets during the process of biooxidation. However, complete biodegradation was not possible with this microorganism. All these studies demonstrate the potential, especially of microorganisms isolated from (poly)aromatic-contaminated soils, to degrade complex graphene materials.

**Fig 2:**
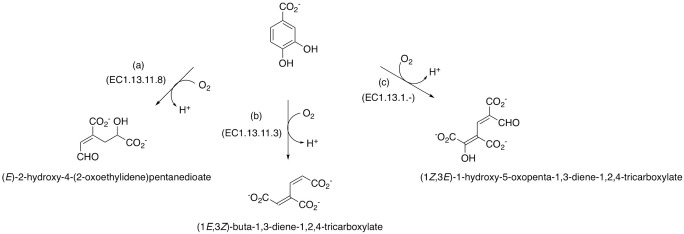
Initial step reactions of protocatechuic acid cleavage by aerobes: (a) *meta*-cleavage, (b) *ortho*-cleavage, and (c) *para*-cleavage.

Some aerobic bacteria are also able to use GO and other GBMs as single carbon sources. They transform the carbon nanomaterials by extracellular reduction of hydrogen peroxide coupled to the respiration of Fe(III), thus producing hydroxyl free radicals that attack the structure of GO (15). Other plausible pathways of GO aerobic degradation include the formation of dihydrodiol intermediates that can be further subjected to *ortho-* or *meta-*cleavage of the attacked carbon ring(s), leading to the formation of structures similar to PAHs, with downstream intermediates such as protocatechuates and catechols (16–18). Protocatechuic acid can be degraded via three known pathways, all of which were described in aerobic bacteria: (i) *meta*-cleavage pathway, with initial enzyme protocatechuate 4,5-dioxygenase (EC 1.13.11.8) leading to the formation of 4-carboxy-2-hydroxymuconate-6-semialdehyde, which at the end is degraded to oxaloacetate and pyruvate before entering the tricarboxylic acid (TCA) cycle (16); (ii) *ortho*-cleavage or 3-oxoadipate pathway, with central enzyme protocatechuate 3,4-dioxygenase (EC 1.13.11.3), where the 3-carboxy-*cis*,*cis*-muconate is further degraded to 3-oxoadipate and the latter one to acetyl-CoA which enters TCA (17); and (iii) *para*-cleavage, with central enzyme protocatechuate 2,3-dioxygenase (EC 1.13.11.2) and formation of (2*Z*,4*Z*)-2-hydroxy-5-carboxymuconate-6-semialdehyde that further is degraded to (2*Z*)-2-hydroxypenta-2,4-dienoate, acetaldehyde, and pyruvate further entering TCA cycle (18). Initial and benzene ring opening reactions of all three pathways are shown in Fig. 2. In addition to these pathways, aerobic bacteria were reported to assimilate many different aromatic hydrocarbons, where the aromatic ring is cleaved.

Aerobic bacteria attack the resonance-stabilized benzene ring oxidatively by introducing hydroxyl groups and by subsequent oxygenolytic benzene ring cleavage. These reactions are exergonic and require molecular oxygen as a co-substrate.

### Impact on anaerobic bacteria

No studies on anaerobic interactions of bacteria with GO and GBMs or their degradation have been documented yet. Nevertheless, scientific information on anaerobic PAH degradation demonstrates plausible bacterial metabolic pathways that might apply to GO and GBMs. In absence of oxygen, numerous bacteria can degrade PAHs; however, anaerobic degradation processes are not as energy-efficient for the bacterial cells as compared to aerobic processes. Nonetheless, the environmental importance of slowly anaerobically processing recalcitrant pollutants in waters, sediments, or soils has to be seriously considered.

In contrast to the aerobes, the use of O_2_-dependent mono- and dioxygenases is irrelevant under anoxic conditions. Anaerobic bacteria use mainly reductive reactions for the dearomatization of the benzene nucleus, and these reactions are far more difficult to achieve, in contrast to oxidative processes. Therefore, anaerobic bacteria have evolved strategies to degrade aromatic compounds using varied metabolic pathways, some of which are still not yet fully understood. The overall strategy is to convert the aromatic substrates into a few key intermediates which are substrates for the reductases. When PAHs and aromatic compounds are anaerobically degraded, the central metabolites are thioesters of benzoic acid (benzoyl-CoA) or hydroxybenzoic acid. As alternative final electron acceptors, bacteria use sulfates, sulfites, thiosulfates, elemental sulfur, nitrate and nitrite ions, oxidized metal (e.g., Fe^3+^, Mn^4+^) or metalloid ions (e.g., arsenate or selenate), and bicarbonate ions or protons (7).

The most well-studied reactions are (i) the activation of toluene by the addition of co-substrate fumarate to form (*R*)-benzylsuccinate. The reaction is catalyzed by the O_2_-sensitive toluene-activating benzyl succinate synthase (EC 4.1.99.11), a glycyl radical enzyme; (ii) the O_2_-independent hydroxylation of ethylbenzene to give (*S*)-1-phenylethanol catalyzed by ethylbenzene dehydrogenase (EBDH) (EC 1.17.99.2). EBDH showed the ability to enantioselectively catalyze the same reaction to over 30 other ring-substituted mono- and bicyclic aromatic compounds to the respective alcohols with (*S*)-configuration (19); (iii) the complete oxidation of 4-alkylbenzoates to CO_2_, via specific 4-methylbenzoyl-CoA pathway in addition to the classical ATP-dependent benzoyl-CoA degradation pathway; (iv) decarboxylation of 4-hydroxyphenylacetate to *p-*cresol, further cleaved to form 3-hydroxypimelyl-CoA (19); and (v) degradation of benzene and naphthalene, via carboxylation to benzoate and 2-naphthoate, which are then decarboxylated by carboxylase-like putative proteins (20). These activation and reduction reactions lead to the formation of central intermediate benzoyl-CoA (19).

In anaerobes, diverse metabolic pathways are used to cleave the benzoyl-CoA and its further assimilation in bacterial cells (19). First, in the central benzoyl-CoA pathway, the dearomatization of benzoyl-CoA proceeds via an ATP-dependent activation to form cyclohex-1,5-diene-1-carbonyl-CoA, which is downstream hydroxylated to 3-hydroxypimeloyl-CoA. For this pathway, one characteristic is the presence of an ATP-dependent, oxygen-sensitive benzoyl-CoA reductase (BCR) Class I enzyme (EC 1.3.7.8.) (21). This pathway is predominantly abundant among facultative anaerobes. Second, the obligate anaerobes, in contrast, activate benzoyl-CoA in an ATP-independent reaction. In this pathway, the intermediates are further β-oxidized to acetyl-CoA and CO_2_. The characteristic enzyme for this pathway is the bifurcating BCR Class II enzyme (EC 1.3.1 .M30), highly oxygen-sensitive, which is not present among aerobes (19, 22). The reaction mechanism of the BCR Class II enzyme is still not completely decrypted. The third metabolic pathway involves a new type of arylcarboxyl-CoA reductases (ATP-independent and oxygen-insensitive), recently discovered in sulfate-reducing naphthalene degraders and is only known for anaerobic PAH degradation (23).

### Bioflotation: “green” separation of particles

Although complete microbial degradation of graphene nanomaterials is one way to avoid the release of hazardous compounds into the environment, recovery and reuse of these compounds could be a better and more sustainable alternative. Microorganisms and metabolites have been successfully applied in bioleaching of metals. More recently and according to recent fundamental studies, bioflotation and bioflocculation emerging technologies have proved to be effective in the beneficiation of these minerals. Indeed, several bacteria, metabolites, and biomolecules such as excreted proteins and polysaccharides are able to significantly alter the mineral surface chemical properties. Therefore, the bacterial cells and their metabolites can be used to selectively separate minerals by flotation processes (24).

Typically, flotation is a simple separation technology originally applied in the mineral industry to extract valuable minerals from ores. However, compared to classical processes which require toxic chemicals, bioreagents can be a promising green alternative.

Qu et al. monitored the increased formation of extracellular polymeric substances (EPS) when the strain *Labrys* sp. WJW was incubated with GO materials (11). EPS are well known to mediate the attachment of bacterial cells to surfaces and biofilm formation (25). Their binding affinities to varied surfaces make them interesting for their application in flotation processes. Consequently, several studies investigated the effect of EPS as a bioreagent in mineral separation. Cells, EPS, or other compounds that interact with the surface of GO particles could be attractive “green” flotation agents for GO particle separation and recycling processes in the future.

### Conclusion

In recent years, the carbon allotrope graphene has emerged as one of the most promising materials. While in some studies, it was reported that graphene can persist in the environment and potentially disrupt microbial communities, others suggest that it may be slowly biodegradable. Thanks to a variety of catalytic reactions that are already known, under study, or as yet unknown that are involved in the degradation of other recalcitrant or toxic substances, such as PAHs and other aromatic compounds, we suggest that GO can be subjected to biodegradation.

In any case, the interaction of graphene with the environment and its bacterial degradation under various conditions remain largely unexplored, and further investigations are urgently needed.

## References

[1] Beloin-Saint-Pierre D, Hischier R. 2021. Towards a more environmentally sustainable production of graphene-based materials. Int J Life Cycle Assess 26:327–343. doi:10.1007/s11367-020-01864-z

[2] Braylé P, Pinelli E, Gauthier L, Mouchet F, Barret M. 2022. Graphene-based nanomaterials and microbial communities: a review of their interactions, from ecotoxicology to bioprocess engineering perspectives. Environ Sci Nano 9:3725–3741.

[3] Guo S, Garaj S, Bianco A, Ménard-Moyon C. 2022. Controlling covalent chemistry on graphene oxide. Nat Rev Phys 4:247–262. doi:10.1038/s42254-022-00422-w

[4] Achawi S, Pourchez J, Feneon B, Forest V. 2021. Graphene-based materials in vitro toxicity and their structure-activity relationships: a systematic literature review. Chem Res Toxicol 34:2003–2018. doi:10.1021/acs.chemrestox.1c00243 34424669

[5] Yadav S, Singh Raman AP, Meena H, Goswami AG, Kumar V, Jain P, Kumar G, Sagar M, Rana DK, Bahadur I, Singh P. 2022. An update on graphene oxide: applications and toxicity. ACS Omega 7:35387–35445. doi:10.1021/acsomega.2c03171 36249372 PMC9558614

[6] Döscher H, Reiss T. 2021. Graphene roadmap briefs (No.1): innovation interfaces of the graphene flagship. 2D Mater 8:022004. doi:10.1088/2053-1583/abddcc

[7] Patel AB, Shaikh S, Jain KR, Desai C, Madamwar D. 2020. Polycyclic aromatic hydrocarbons: sources, toxicity, and remediation approaches. Front Microbiol 11:562813. doi:10.3389/fmicb.2020.562813 33224110 PMC7674206

[8] Chung H, Kim MJ, Ko K, Kim JH, Kwon H-A, Hong I, Park N, Lee S-W, Kim W. 2015. Effects of graphene oxides on soil enzyme activity and microbial biomass. Sci Total Environ 514:307–313. doi:10.1016/j.scitotenv.2015.01.077 25668283

[9] Kim M-J, Ko D, Ko K, Kim D, Lee J-Y, Woo SM, Kim W, Chung H. 2018. Effects of silver-graphene oxide nanocomposites on soil microbial communities. J Hazard Mater 346:93–102. doi:10.1016/j.jhazmat.2017.11.032 29248800

[10] Forstner C, Orton TG, Skarshewski A, Wang P, Kopittke PM, Dennis PG. 2019. Effects of graphene oxide and graphite on soil bacterial and fungal diversity. Sci Total Environ 671:140–148. doi:10.1016/j.scitotenv.2019.03.360 30928743

[11] Qu Y, Wang J, Ma Q, Shen W, Pei X, You S, Yin Q, Li X. 2018. A novel environmental fate of graphene oxide: biodegradation by a bacterium Labrys sp. WJW to support growth. Water Res. 143:260–269. doi:10.1016/j.watres.2018.03.070 29986236

[12] Liu L, Zhu C, Fan M, Chen C, Huang Y, Hao Q, Yang J, Wang H, Sun D. 2015. Oxidation and degradation of graphitic materials by naphthalene-degrading bacteria. Nanoscale 7:13619–13628. doi:10.1039/c5nr02502h 26205788

[13] Zhang L, Petersen EJ, Habteselassie MY, Mao L, Huang Q. 2013. Degradation of multiwall carbon nanotubes by bacteria. Environ Pollut 181:335–339. doi:10.1016/j.envpol.2013.05.058 23859846

[14] Zhu C, Liu L, Fan M, Liu L, Dai B, Yang J, Sun D. 2014. Microbial oxidation of graphite by Acidithiobacillus ferrooxidans CFMI-1. RSC Adv 4:55044–55047. doi:10.1039/C4RA09827G

[15] Wang J, Ma Q, Zhang Z, Li S, Diko CS, Dai C, Zhang H, Qu Y. 2020. Bacteria mediated fenton-like reaction drives the biotransformation of carbon nanomaterials. Sci Total Environ 746:141020. doi:10.1016/j.scitotenv.2020.141020 32750576

[16] DAGLEY S, PATEL MD. 1957. Oxidation of p-cresol and related compounds by a Pseudomonas. Biochem J 66:227–233. doi:10.1042/bj0660227 13445676 PMC1199997

[17] Harwood CS, Parales RE. 1996. The beta-ketoadipate pathway and the biology of self-identity. Annu Rev Microbiol 50:553–590. doi:10.1146/annurev.micro.50.1.553 8905091

[18] Crawford RL. 1975. Novel pathway for degradation of protocatechuic acid in Bacillus species. J Bacteriol 121:531–536. doi:10.1128/jb.121.2.531-536.1975 163224 PMC245963

[19] Rabus R, Boll M, Heider J, Meckenstock RU, Buckel W, Einsle O, Ermler U, Golding BT, Gunsalus RP, Kroneck PMH, Krüger M, Lueders T, Martins BM, Musat F, Richnow HH, Schink B, Seifert J, Szaleniec M, Treude T, Ullmann GM, Vogt C, von Bergen M, Wilkes H. 2016. Anaerobic microbial degradation of hydrocarbons: from enzymatic reactions to the environment. J Mol Microbiol Biotechnol 26:5–28. doi:10.1159/000443997 26960061

[20] Meckenstock RU, Boll M, Mouttaki H, Koelschbach JS, Cunha Tarouco P, Weyrauch P, Dong X, Himmelberg AM. 2016. Anaerobic degradation of benzene and polycyclic aromatic hydrocarbons. J Mol Microbiol Biotechnol 26:92–118. doi:10.1159/000441358 26960214

[21] Boll M. 2005. Dearomatizing benzene ring reductases. J Mol Microbiol Biotechnol 10:132–142. doi:10.1159/000091560 16645310

[22] Boll M, Einsle O, Ermler U, Kroneck PMH, Ullmann GM. 2016. Structure and function of the unusual tungsten enzymes acetylene hydratase and class II benzoyl-coenzyme A reductase. J Mol Microbiol Biotechnol 26:119–137. doi:10.1159/000440805 26959374

[23] Eberlein C, Estelmann S, Seifert J, von Bergen M, Müller M, Meckenstock RU, Boll M. 2013. Identification and characterization of 2-naphthoyl-coenzyme A reductase, the prototype of a novel class of dearomatizing reductases. Mol Microbiol 88:1032–1039. doi:10.1111/mmi.12238 23646996

[24] Kinnunen P, Miettinen H, Bomberg M. 2020. Review of potential microbial effects on flotation. Minerals 10:533. doi:10.3390/min10060533

[25] Kinzler K, Gehrke T, Telegdi J, Sand W. 2003. Bioleaching - a result of interfacial processes caused by extracellular polymeric substances (EPS). Hydrometallurgy 71:83–88. doi:10.1016/S0304-386X(03)00176-2

